# Stage-Dependent Predation by *Scymnus* (*Scymnus*) *folchinii* Against *Myzus persicae*: Functional Response and First-Instar Prey Sharing

**DOI:** 10.3390/insects17060629

**Published:** 2026-06-15

**Authors:** Yu-Cheng Fang, Xiao-Li Mao, Yang Zhang, Xin-Yi Wang, Tong-Xian Liu, Yi Feng

**Affiliations:** 1 State Key Laboratory for Crop Stress Resistance and High-Efficiency Production, Key Laboratory of Integrated Pest Management on Crops in Northwestern Loess Plateau of Ministry of Agriculture and Rural Affairs, College of Plant Protection, Northwest A&F University, Yangling 712100, China; fangyucheng@nwafu.edu.cn (Y.-C.F.); mxiaoli@nwafu.edu.cn (X.-L.M.); zhangyang4673@nwafu.edu.cn (Y.Z.); 2024010352@nwafu.edu.cn (X.-Y.W.); 2 Institute of Entomology, Guizhou University, Guiyang 550025, China; tx.liu@gzu.edu.cn

**Keywords:** biological control, Coccinellidae, functional response, green peach aphid, feeding behavior, prey sharing, Scymnini, integrated pest management

## Abstract

Small scymnine coccinellids, including many *Scymnus* species, are common aphid predators, but stage-specific feeding data are still limited for many species. We investigated feeding behavior, first-instar prey sharing, stage-specific consumption, and functional responses of *Scymnus (Scymnus) folchinii* (Canepari) on third-instar *Myzus persicae* nymphs on chili pepper seedlings. Larvae showed feeding behavior consistent with extra-oral digestion, whereas adults consumed aphids by direct mastication. In the single-prey first-instar assay, prey sharing occurred in all 15 arenas; the period during which two or more larvae fed on the same aphid lasted a median of 31.0 min and accounted for 84.8% of the observed feeding period. Aphid consumption varied with predator stage, exposure time, and initial aphid density. At the highest tested density of 16 aphids, third- and fourth-instar larvae and adults left few aphids alive after 24 h in the seedling micro-arena. Logistic regression diagnosed Type II responses only for adults at 1 h, whereas AIC-based model comparison selected Rogers’ Type II in 15 of 18 stage-by-duration combinations. Short exposures indicated higher attack rates and shorter handling times in fourth instars and adults, whereas longer exposures were affected by prey depletion and satiation. These results show clear stage dependence in aphid consumption by *S. folchinii* and justify further testing on larger plants and under field conditions.

## Simple Summary

Small ladybird beetles of the genus *Scymnus* occur in many crop systems, but their feeding capacity is less often measured than that of larger aphid-feeding ladybirds. We examined how larvae and adults of *Scymnus (Scymnus) folchinii* fed on third-instar green peach aphids on chili pepper seedlings. Larvae and adults handled aphids differently. Larvae showed feeding behavior consistent with extra-oral digestion, whereas adults chewed aphids directly. In a single-prey group-feeding assay, first-instar larvae often fed on the same aphid at the same time. Aphid consumption increased from early larvae to late larvae and adults. Functional-response models most often fitted the data as Type II-like responses, but the response type was not clearly resolved for every stage and exposure period. Late larvae and adults removed aphids most rapidly in the seedling arena. These laboratory results support further testing of this small ladybird under field conditions.

## 1. Introduction

Aphids are persistent pests in vegetable and field-crop systems because they reproduce rapidly, colonize plants quickly, and readily develop resistance to multiple insecticide classes. The green peach aphid, *Myzus persicae* (Sulzer), is particularly important in Solanaceae crops because it damages plants directly through phloem feeding and indirectly as a virus vector. These traits make *M. persicae* a persistent target in integrated pest management, especially in systems where insecticide resistance or residue concerns limit repeated chemical use [1,2,3,4].

Predatory ladybird beetles (Coleoptera: Coccinellidae) are among the most important aphidophagous natural enemies in agroecosystems. Both larvae and adults can consume large numbers of aphids, but their impact is not uniform across the life cycle because body size, mobility, sensory capacity, digestive capacity, and reproductive demand change during development [5,6]. Evaluations based only on adults can therefore give an incomplete estimate of the predatory capacity of a coccinellid population.

Research on aphidophagous coccinellids has historically emphasized larger and more conspicuous species, whereas minute scymnine coccinellids, including many *Scymnus* species with adult body lengths of approximately 1–3 mm, remain less studied despite their frequent occurrence and potential numerical importance in natural enemy communities [6,7,8,9]. This may be important because mass-specific metabolic rate generally increases as body size decreases; therefore, small predators may have relatively high energetic demands per unit biomass and may contribute to early aphid-colony exploitation [10]. Field monitoring has also documented diverse coccinellid and syrphid assemblages in crop systems [11,12]. These records are useful, but they do not by themselves show how small predators could contribute to aphid management. This matters because small predators may use different parts of the plant, encounter early aphid colonies, or persist when larger predators are scarce.

Among these small-bodied predators, aphidophagous Scymnini are repeatedly sampled, but relatively few species have been tested experimentally as biological-control agents [13,14]. The genus *Scymnus* Kugelann includes more than 800 described species. Although *Scymnus* taxonomy has been investigated [15,16,17], predation behaviors are still unknown for many species. Existing biological studies, such as work on *S. frontalis*, indicate that basic stage-specific predation data are still lacking for this genus [18]. *Scymnus* (*Scymnus) folchinii* (Canepari) is recorded from China and is commonly encountered in aphid-infested crop habitats [11,12]. For *S. folchinii*, several aspects remain unclear: stage-specific foraging behavior from larvae to adults, whether early-instar larvae can feed simultaneously on one aphid, and how consumption changes with aphid density.

Functional response analysis relates prey consumption to prey density and is commonly used to compare predator performance under controlled conditions [19,20,21,22]. Functional response type and parameters such as attack rate and handling time are often used to infer predator efficiency, potential prey suppression, and likely limitations imposed by handling, digestion, and satiation [20,21,22,23,24]. However, functional response classification can be sensitive to model choice, prey depletion, exposure duration, and predator stage. For this reason, logistic diagnosis of response shape should be separated from AIC-based comparison among depletion models when interpreting laboratory data [24,25,26].

Functional-response studies on aphidophagous coccinellids are extensive, and Type II responses have frequently been reported for ladybird predators [27,28]. For example, *Coccinella undecimpunctata* exhibits a Type II functional response to *M. persicae*, with fourth-instar larvae showing a shorter handling time than adults [27]. Related work on the same predator has also examined the voracity of fourth-instar larvae and adults when feeding on aphids and whiteflies [28]. These studies show that functional-response parameters can vary with predator stage, prey species, and experimental conditions [27,28]. However, for small scymnine coccinellids, especially many *Scymnus* species, stage-specific feeding behavior, early-instar prey sharing, and functional-response parameters on host plants remain poorly documented.

We therefore examined three aspects of *S. folchinii* predation on *M. persicae*: larval and adult feeding behavior, prey sharing by first-instar larvae, and stage-specific functional responses. We tested the hypotheses that (i) feeding mode differs between larval and adult stages; (ii) first-instar larvae can exploit aphids simultaneously under group-feeding conditions; (iii) predatory capacity increases from early larvae to late larvae and adults; and (iv) functional responses are predominantly Type II-like but vary with developmental stage and exposure duration. By combining behavioral observations, stage-specific consumption measurements and functional-response analyses, this study provides baseline biological information on the predatory capacity of *S. folchinii*, a small scymnine coccinellid that has not been experimentally characterized. These findings provide a basis for future evaluation under more realistic greenhouse or field conditions.

## 2. Materials and Methods

### 2.1. Plant Material, Insect Colonies, and Species Identification

Chili pepper plants (*Capsicum annuum* L., var. Shulahuojian F1) were used for maintaining the *M. persicae* colony. Fresh chili pepper leaves were used in the prey-sharing assay, whereas young seedlings at the first true-leaf stage were used in the aphid-consumption and functional-response assays. Colonies of *M. persicae* and *S. folchinii* were established from individuals collected in July 2024 from chili pepper and corn fields at the Experimental Farm of Northwest A&F University, Yangling, Shaanxi, China (34°17′ N, 108°04′ E). Adult *S. folchinii* were identified using the illustrated guide to Chinese ladybird beetles [18] and the taxonomic revision of Chinese *Scymnus* by Chen et al. [16]. The species identity was further checked against a previous survey identification assisted by Guo-Yue Yu, Beijing Academy of Agriculture and Forestry Sciences, based on diagnostic morphological images and characters. Voucher specimens from the colony were deposited in the Key Laboratory of Integrated Pest Management on Crops in Northwestern Loess Plateau, College of Plant Protection, Northwest A&F University, Yangling, China. *M*. *persicae* was maintained on potted chili pepper plants in a climate-controlled insectary.

Adult *S. folchinii* were initially maintained in 3.5 cm Petri dishes and supplied ad libitum with *M. persicae*. Adult sex was first assessed using pronotal coloration, because females usually had a black pronotum or orange coloration restricted to the margins, whereas males generally had a larger orange area on the pronotum. However, pronotal coloration was not fully reliable, as some males also had a predominantly black pronotum. Therefore, coloration was used only as a preliminary cue, and adult sex was confirmed behaviorally. Several adults were placed together and observed until copulation occurred; mating pairs were then separated immediately, and sex was assigned according to copulatory position. Only adults whose sex could be assigned unambiguously were used in sex-specific assays. For colony propagation and production of experimental larvae, confirmed mating pairs were transferred to new dishes for oviposition. Eggs were collected daily, and newly hatched larvae were reared individually in 3.5 cm Petri dishes with excess aphids until pupation or until they reached the required instar for experiments. First-instar larvae from these synchronized cohorts were used in the prey-sharing assay, whereas healthy and active first-, second-, third-, and fourth-instar larvae were selected for the stage-specific consumption and functional-response assays. Newly emerged adults were collected within 24 h of emergence, maintained individually under standardized conditions, and used in adult experiments. All insect colonies and experiments were maintained at 25 ± 1 °C, 65 ± 5% RH, and a 16:8 h light:dark photoperiod.

### 2.2. Foraging Behavior Observation

Foraging behavior was observed and recorded during colony maintenance and during preparation of experimental insects. Larvae of different instars and adults were observed feeding on aphids on host plants using a stereomicroscope or hand lens. Feeding events were photographed for each larval instar and for adult females and males when possible. These observations were used only to describe feeding mode; they were not treated as a replicated behavioral experiment.

### 2.3. Prey-Sharing Assay

During colony maintenance, we occasionally observed more than one early-instar larva feeding on the same aphid. We therefore used a constrained single-prey group-feeding assay to test whether first-instar larvae could share a single aphid under prey-limited conditions. This assay was designed as a behavioral-capacity test rather than as a simulation of natural aphid-colony conditions. Each arena consisted of a 3.5 cm Petri dish containing a fresh chili pepper leaf disk placed on 1% agar to maintain leaf freshness and humidity. One third-instar *M. persicae* nymph was introduced onto the leaf disk and allowed to settle for 5 min. Four first-instar larvae were then gently introduced into the arena and observed continuously for 120 min under a stereomicroscope. For each arena, the initiation and cessation times of feeding by the first, second, third, and fourth larvae were recorded. Larvae were assigned to feeding order according to the sequence in which they began feeding, rather than by pre-assigned individual identity. Feeding initiation was defined as sustained feeding on the aphid, and feeding cessation was defined as detachment from the prey without immediate resumption. Prey sharing was defined as temporal overlap in feeding by two or more larvae on the same aphid. We therefore quantified overlap in feeding time, rather than simply counting how many larvae eventually contacted the aphid. The assay was replicated 15 times, with each arena treated as one independent replicate.

### 2.4. Stage-Specific Consumption and Functional Response Experiment

Third-instar *M. persicae* nymphs were used to standardize prey size and eliminate confounding from aphid reproduction. To obtain synchronized cohorts, ten adult aphids were placed on a 3.5 cm chili pepper leaf disk positioned on 1% agar in a 3.5 cm Petri dish. After 24 h, adults were removed and nymphs were reared until the third instar.

Functional response experiments were conducted in a controlled micro-arena consisting of a vertical borosilicate glass tube (inner diameter 2.0 cm, height 10.0 cm) enclosing a single chili pepper seedling (at the first true-leaf stage). The root system was connected to a water reservoir through absorbent cotton to maintain plant turgor, while a dry cotton barrier reduced excess humidity in the aerial portion. The tube top was sealed with a ventilated cap fitted with fine nylon mesh (120 μm pore size) to allow gas exchange and prevent insect escape.

Five initial aphid densities were tested: 1, 2, 4, 8, and 16 third-instar nymphs. The maximum density of 16 aphids was selected based on preliminary observations showing that adult *S. folchinii* consumed approximately 15 third-instar *M. persicae* nymphs within 24 h under comparable micro-arena conditions. Thus, the density range was intended to cover the range from low prey availability to near-saturating prey availability while avoiding excessive crowding on first true-leaf chili pepper seedlings. Aphids were introduced onto the plant and allowed to acclimate for 1 h before predator introduction. Healthy, active first- to fourth-instar larvae and 2–3-day-old adults of confirmed sex were selected as predators. Before the experiment, predators were individually held in 3.5 cm Petri dishes for 12 h with a water-saturated cotton ball to standardize hunger. This starvation period was applied consistently across predator stages to reduce variation caused by recent feeding history, but it may have affected first-instar larvae more strongly than later instars or adults because of their smaller body size and limited energetic reserves. One larva or adult was then introduced into the center of each arena. Cumulative aphid consumption was recorded after 1, 6, and 24 h, and predators were removed after 24 h. Each larval stage × aphid-density treatment was replicated 10 times. Adult treatments were replicated separately by sex, with 10 adult females and 10 adult males tested at each aphid density.

Predator-free control arenas were established for each initial aphid density to estimate background aphid mortality. Control arenas contained the same chili pepper seedling micro-arena and the same initial aphid densities as predator treatments, but no predator was introduced. Each aphid-density control was replicated 6–8 times. Aphids were checked after 24 h, and the number of surviving aphids was recorded.

All colonies and experiments were maintained at 25 ± 1 °C, 65 ± 5% relative humidity, and a 16:8 h light:dark photoperiod.

### 2.5. Data Analysis

#### 2.5.1. Prey-Sharing Analysis

For temporal recruitment into feeding, initiation times were recorded by feeding order. Larvae that did not initiate feeding within 120 min were treated as right-censored at 120 min. Cumulative feeding-initiation curves were generated using Kaplan–Meier estimates and used descriptively to show the timing with which additional larvae joined feeding. For each arena, feeding intervals were reconstructed from initiation and cessation times. Sharing duration was calculated as the total duration during which at least two larvae fed simultaneously. Because each arena was an independent replicate, sharing duration was summarized at the arena level using the median, interquartile range, and observed range. A bootstrap 95% confidence interval for the median sharing duration was estimated using 10,000 resamples.

#### 2.5.2. Aphid Consumption Models

Cumulative aphid consumption at 1, 6, and 24 h was analyzed using a repeated-measures beta-binomial mixed model with predator identity as a random intercept. Because aphid survival in predator-free controls was high after 24 h, no background-mortality correction was applied to the predator-treatment consumption data. Predator stage, exposure time, initial aphid density, and their interactions were included as fixed effects; initial aphid density was treated as a log_2_-transformed continuous predictor. Twenty-four-hour proportional consumption was analyzed using beta-binomial endpoint models. Adult female and male consumption was additionally compared using a separate adult-only endpoint model. Interval-specific aphid consumption for 0–1 h, 1–6 h, and 6–24 h was analyzed using a conditional beta-binomial mixed model with predator identity as a random intercept. Type III Wald χ^2^ tests were used for fixed effects.

#### 2.5.3. Functional Response Diagnosis and Mechanistic Model Fitting

Functional response type was first diagnosed using logistic regression of the proportion of prey consumed, $N_{e}/N_{0}$, as a function of initial prey density, $N_{0}$, following Juliano [24]. A binomial generalized linear model with a logit link was fitted as

(1) $$logit\left(N_{e}/N_{0}\right)=P_{0}+P_{1}N_{0}+P_{2}N_{0}^{2}+P_{3}N_{0}^{3}.$$

Here, $N_{e}$ is the number of prey consumed, $N_{0}$ is the initial prey density, and $P_{0}$, $P_{1}$, $P_{2}$, and $P_{3}$ are the intercept, linear, quadratic, and cubic coefficients, respectively. A significantly negative $P_{1}$ was interpreted as evidence of a Type II functional response because it indicates a decline in the proportion of prey consumed with increasing prey density. A significantly positive $P_{1}$, followed by a negative quadratic coefficient, $P_{2}$, was interpreted as evidence of a Type III functional response. When the linear coefficient was not significant, the functional response type was considered not resolved by logistic regression. This diagnostic step was used only to assess response shape and was not used for mechanistic parameter estimation.

Because prey were not replaced during the experiment, mechanistic models that account for prey depletion were fitted using maximum likelihood estimation. Four candidate models were fitted separately to each predator stage × exposure duration dataset. The Rogers’ random predator equation was used as the standard Type II depletion model:

(2) $$N_{e}=N_{0}\{1-exp\left[-a\left({T-T}_{h}N_{e}\right)\right]\}.$$

Here, a is the attack rate, $T_{h}$ is the handling time, and T is the total exposure duration. Because $N_{e}$ appears on both sides of the equation, this model was solved numerically during model fitting.

A generalized Rogers’ model with a scaling exponent was also fitted. In this model, attack rate was allowed to vary with prey density according to

(3) $$a\left(N_{0}\right)=bN_{0}^{q},$$

giving

(4) $$N_{e}=N_{0}\{1-exp\left[-bN_{0}^{q}\left({T-T}_{h}N_{e}\right)\right]\}.$$

Here, b is the search coefficient and q is the scaling exponent describing density dependence in attack rate. When q=0, the model reduces to the standard Rogers’ Type II equation. Therefore, this model was interpreted as a generalized density-dependent attack-rate model rather than as a discrete Type III model.

The Hassell Type III model without replacement was fitted by allowing the attack coefficient to vary with initial prey density as

(5) $$a\left(N_{0}\right)=bN_{0}/\left(1+cN_{0}\right)$$

where *a*(*N*_0_) is the density-dependent attack coefficient, *N*_0_ is the initial prey density, and *b* and *c* are fitted constants describing the increase and saturation of attack rate with prey density. This density-dependent attack coefficient was then incorporated into the Rogers non-replacement framework, giving

(6) $$N_{e}=N_{0}\{1-exp\left[-(bN_{0}/(1+cN_{0}))\left({T-T}_{h}N_{e}\right)\right]\},$$

where *N_e_* is the number of prey consumed, *T* is the exposure duration, and *T_h_* is handling time. Because *N_e_* appears on both sides of the equation, the model was solved numerically during maximum-likelihood fitting.

The Holling Type I model was fitted as

(7) $$N_{e}=aTN_{0}.$$

Here, a is the constant attack rate and T is the exposure duration. Although this model is biologically simple and does not include handling time, it was retained as a low-complexity candidate model for comparison.

Model performance was compared using Akaike’s information criterion, calculated as

(8) $${AIC}_{i}=2k_{i}-2ln\left({\hat{L}}_{i}\right).$$

Here, $k_{i}$ is the number of estimable parameters in model i, and ${\hat{L}}_{i}$ is the maximized likelihood. For each predator stage and exposure duration, AIC differences were calculated as

(9) $$\Delta_{i}={AIC}_{i}-{AIC}_{min},$$

where ${AIC}_{min}$ is the lowest AIC among the candidate models. Akaike weights were then calculated as

(10) $$w_{i}=exp\left(-0.5\Delta_{i}\right)/\sum_{r=1}^{R}exp\left(-0.5\Delta_{r}\right),$$

where R is the number of candidate models. The model with the lowest AIC was considered the best-supported model. Models with $\Delta_{i}<2$ were considered to have substantial support and were interpreted as evidence of model-selection uncertainty rather than clear support for a single functional response form.

For the Rogers’ Type II model, attack rate, a, and handling time, $T_{h}$, were estimated for each predator stage and exposure duration. Because the Rogers’ Type II model provides directly interpretable biological parameters and was the most frequently supported model in the dataset, its a and $T_{h}$ estimates were used as the primary functional response parameters for stage-by-duration comparison. For the generalized Rogers’ model, the scaling exponent, q, was estimated to assess whether attack rate changed with prey density. Values of q close to zero indicate no detectable density dependence in attack rate, positive values indicate increasing attack rate with prey density, and negative values indicate decreasing attack rate with prey density.

Parameter uncertainty was quantified using non-parametric bootstrapping with 2000 iterations. Bootstrap 95% confidence intervals were calculated for attack rate, handling time, and the scaling exponent where applicable. Parameter estimates were interpreted cautiously when confidence intervals were wide or when competing models had similar AIC support. Logistic regression diagnosis, AIC-based model comparison, Rogers’ Type II parameter estimates, and generalized Rogers’ scaling exponents were reported separately to avoid conflating functional response diagnosis with mechanistic model selection.

All statistical analyses described above were conducted in R version 4.5.2 (2025-10-31). Functional-response models were fitted using frair version 0.5.203. Beta-binomial mixed models were fitted using glmmTMB version 1.1.14. Kaplan–Meier estimates were generated using survival version 3.8-6. Model tests and diagnostic checks were conducted using car version 3.1-5 and brglm2 version 1.1.0, where applicable. Data handling and visualization were conducted using tidyverse version 2.0.0 and related packages, including dplyr version 1.2.1, ggplot2 version 4.0.3, readr version 2.2.0, purrr version 1.2.2, tibble version 3.3.1, tidyr version 1.3.2, stringr version 1.6.0, scales version 1.4.0, patchwork version 1.3.2, janitor version 2.2.1, and knitr version 1.51.

## 3. Results

### 3.1. Stage-Specific Foraging Behavior

**Figure 1:**
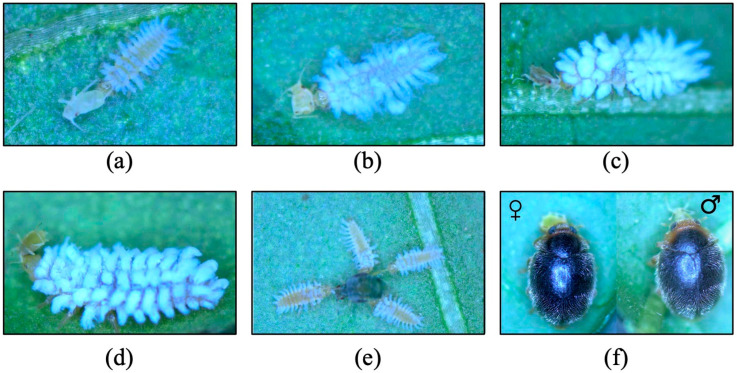
Larval and adult feeding behavior of *S. folchinii* feeding on third-instar *M. persicae* nymphs. (**a**) First-instar larva; (**b**) second-instar larva; (**c**) third-instar larva; (**d**) fourth-instar larva; (**e**) multiple first-instar larvae sharing a single aphid prey; (**f**) adult mastication of an aphid.

Larvae and adults of *S. folchinii* handled aphids differently. Larvae moved over the leaf surface and contacted aphids with their mouthparts. After contacting an aphid, larvae usually bit a leg or antenna (Figure 1a–d) and then showed feeding behavior consistent with extra-oral digestion, including repeated sucking or regurgitation-like movements and the presence of visible fluid at the feeding site. After feeding, empty aphid cuticles were often observed. These observations provide behavioral evidence consistent with extra-oral digestion, but they do not directly confirm the underlying physiological process. In some observations, more than one larva fed on the same aphid, usually from different appendages (Figure 1e). Adults searched the leaf surface with the maxillary palps. When a palp contacted an aphid, the adult usually bit quickly and consumed the prey by mastication (Figure 1f); larger or escaping aphids were restrained with the forelegs. Adults were able to consume the entire aphid body.

### 3.2. Prey Sharing by First-Instar Larvae

Prey sharing occurred in all 15 single-prey arenas (exact binomial 95% CI: 78.2–100.0%). At least two larvae fed on the aphid in every arena. A third larva joined the feeding in 8 of the 15 arenas (53.3%, exact 95% CI: 26.6–78.7%), and a fourth joined in 3 arenas (20.0%, exact 95% CI: 4.3–48.1%). The maximum number of simultaneous feeders varied: two larvae in seven arenas, three larvae in five arenas, and four larvae in three arenas.

**Figure 2:**
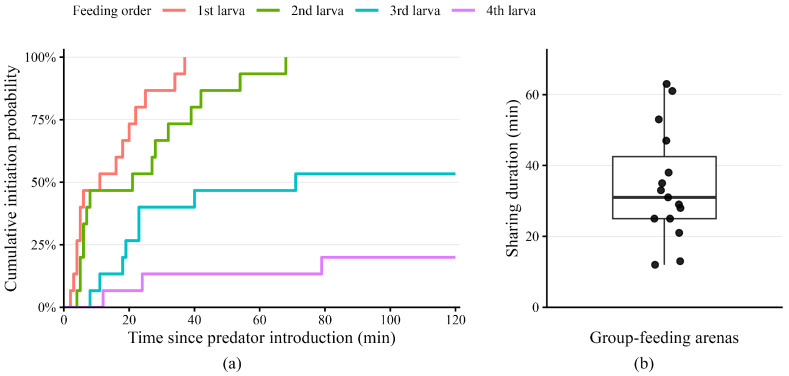
Temporal recruitment into feeding and duration of prey sharing in first-instar *S. folchinii* larvae. (**a**) Cumulative feeding-initiation curves for the first, second, third, and fourth larvae to begin feeding on a single third-instar *M. persicae* nymph during the 120 min observation period. Larvae that did not initiate feeding were right-censored at 120 min. (**b**) Arena-level sharing duration, defined as the total time during which two or more larvae fed simultaneously on the same aphid. Points represent individual arenas; the box shows the median and interquartile range.

The first larva initiated feeding after a median of 11.0 min following predator introduction (IQR: 4.5–21.0 min; range: 2.0–37.0 min). The feeding-initiation curves showed that the first and second larvae fed in all arenas, whereas the third and fourth larvae fed only in a subset of arenas during the 120 min observation period (Figure 2a). The median interval from the first feeding initiation to the onset of prey sharing was 3.0 min (IQR: 1.5–6.5 min; range: 1.0–57.0 min; bootstrap 95% CI for the median: 2.0–5.0 min).

The overall feeding period, from initial feeding to final cessation, lasted a median of 48.0 min (IQR: 33.5–56.0 min; range: 23.0–72.0 min). The median duration of overlapping feeding was 31.0 min per arena (IQR: 25.0–42.5 min; range: 12.0–63.0 min; bootstrap 95% CI for the median: 25.0–38.0 min; Figure 2b). Overlap accounted for a median of 84.8% of the observed feeding period (IQR: 74.6–91.3%; range: 18.1–95.3%). Total larval feeding time, summed across larvae within each arena, reached a median of 86.0 larva-min per arena (IQR: 75.5–101.5 larva-min; range: 46.0–175.0 larva-min).

### 3.3. Aphid Consumption Across Predator Stage, Exposure Time, and Initial Density

In control trials, aphid survival was 100.0%, 100%, 96.9%, 96.4%, and 97.9% at initial densities of 1, 2, 4, 8, and 16 aphids, respectively. Because background mortality was low, no mortality correction was applied to predator-treatment consumption data.

**Figure 3:**
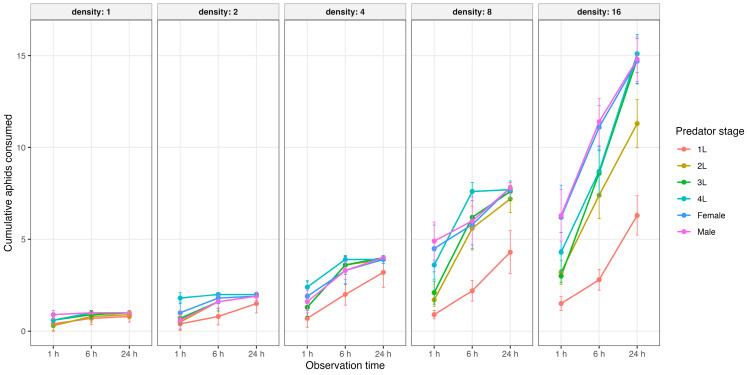
Time-dependent cumulative consumption of third-instar *M. persicae* nymphs by larval and adult *S. folchinii* on chili pepper seedlings across initial aphid densities. Individual predators were exposed to 1, 2, 4, 8, or 16 aphids, and cumulative consumption was recorded after 1, 6, and 24 h. Points and connecting lines show model-estimated cumulative consumption. Separate panels represent initial aphid densities.

Cumulative consumption of third-instar *M. persicae* nymphs differed among predator developmental stages, exposure times, and initial aphid densities (Figure 3; Table 1). In the repeated-measures beta-binomial model, predator stage, exposure time, and initial aphid density all had significant main effects. Significant two-way interactions were detected for predator stage × exposure time, predator stage × initial aphid density, and exposure time × initial aphid density, whereas the three-way interaction was marginal but not significant at α = 0.05. Cumulative consumption increased with time and aphid density, but the pattern differed among predator stages. First-instar larvae consumed the fewest aphids, second instars were intermediate, and third instars, fourth instars, and adults consumed the most. At the highest prey density, late-instar larvae and adults consumed nearly all available aphids after 24 h.

### 3.4. Twenty-Four-Hour Aphid Consumption and Interval-Specific Consumption

Aphid consumption was recorded at 1, 6, and 24 h to characterize temporal feeding dynamics, but the 24 h endpoint was used as the primary measure of short-term predatory capacity. Twenty-four-hour proportional consumption was significantly affected by predator stage and initial aphid density, whereas the stage × density interaction was not significant (Table 1). The negative coefficient for log_2_-transformed initial aphid density (β = −0.567 ± 0.140 SE, z = −4.05, *p* < 0.001) indicated that the proportion of aphids consumed declined as prey density increased, although absolute consumption increased.

**Figure 4:**
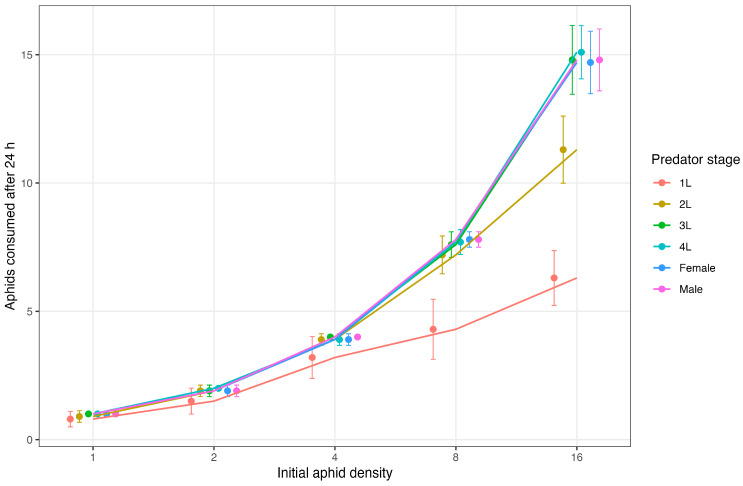
Stage-specific 24 h consumption of third-instar *M. persicae* nymphs by *S. folchinii* on chili pepper seedlings across increasing initial aphid densities. Individual first- to fourth-instar larvae, adult females, and adult males were exposed to 1, 2, 4, 8, or 16 aphids for 24 h. Points and lines show model-estimated numbers of aphids consumed; error bars represent 95% confidence intervals.

At the highest initial density, model-estimated 24 h consumption was 6.53 aphids for first-instar larvae, 11.61 for second-instar larvae, 15.01 for third-instar larvae, 15.15 for fourth-instar larvae, 14.95 for adult females, and 15.00 for adult males (Figure 4). Adult females and males did not differ significantly in 24 h consumption (β = −0.346 ± 0.940 SE, z = −0.37, *p* = 0.713), and the adult sex × density interaction was also not significant (β = 0.0808 ± 0.271 SE, z = 0.30, *p* = 0.765).

Interval-specific consumption differed significantly with predator stage, observation interval, and initial aphid density (Table A1). Significant interactions were detected for predator stage × observation interval, observation interval × initial aphid density, and predator stage × observation interval × initial aphid density, indicating that stage-specific feeding patterns changed across the 0–1 h, 1–6 h, and 6–24 h intervals.

### 3.5. Functional Response Diagnosis and Model Comparison

Logistic regression of proportional prey consumption detected a significantly negative first-order coefficient only in adult predators at the shortest exposure duration. The linear coefficient was significant and negative for adult males at 1 h (P1 = −1.453, z = −2.502, *p* = 0.012) and adult females at 1 h (P1 = −1.242, z = −2.091, *p* = 0.037), indicating Type II responses in these two treatments (Table 2). For all larval stages and for adult treatments at 6 and 24 h, logistic regression did not clearly assign response type; therefore, these combinations were treated as not resolved by logistic regression. No treatment was diagnosed as Type III.

**Figure 5:**
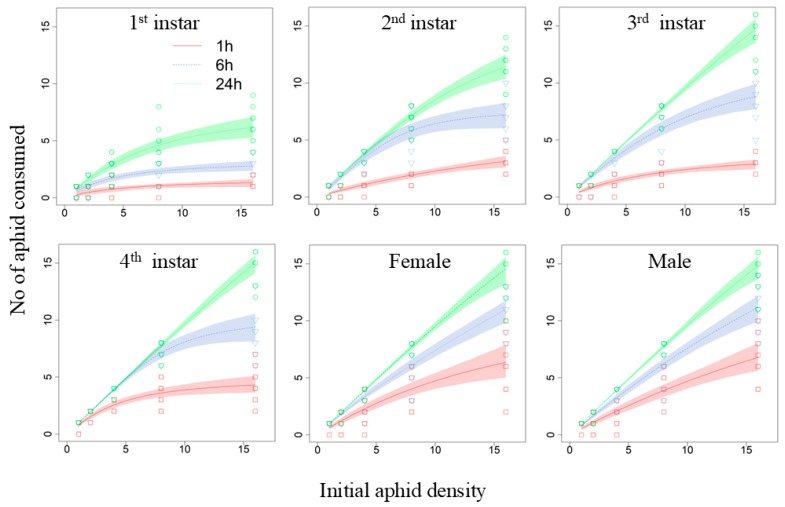
Functional responses of larval and adult *S. folchinii* against third-instar *M*. *persicae* nymphs on healthy chili pepper seedlings over 1, 6, and 24 h. Points indicate observed consumption values, lines represent fitted responses for each exposure duration, and shaded areas are bootstrap 95% confidence intervals based on 2000 bootstrap iterations.

AIC comparison of four mechanistic candidate models showed that the Rogers’ Type II random predator equation was the most frequently supported model. Rogers’ Type II had the lowest AIC in 15 of 18 stage-by-duration combinations. The generalized Rogers’ model with a scaling exponent was selected in three combinations: second-instar larvae at 6 h, adult males at 1 h, and adult females at 6 h. Neither the Hassell Type III model nor the Holling Type I model was selected as the best-supported model in any treatment (Table 2 and Table A2). In several stage-by-duration combinations, the generalized Rogers model had ΔAIC < 2 relative to Rogers’ Type II, indicating model-selection uncertainty. The fitted functional-response curves showed increasing prey consumption with initial aphid density, with stronger saturation at longer exposure durations and in later predator stages (Figure 5).

### 3.6. Attack Rate, Handling Time, and Density-Dependent Scaling

Rogers’ Type II parameter estimates varied strongly among predator stages and exposure durations (Table 3). Because exposure duration was modeled in days, the attack coefficient *a* is reported as d^−1^ for the fixed seedling micro-arena used in this experiment. For readability, handling time *T_h_* was converted from days to minutes per aphid. At 1 h, attack rate was lower in first- and second-instar larvae (a = 9.00 and 8.80 d^−1^, respectively), increased in third-instar larvae (a = 15.38 d^−1^), and reached the highest estimate in fourth-instar larvae (a = 45.63 d^−1^). Adult females and males also showed high 1 h attack rates, with estimates of 24.83 and 19.90 d^−1^, respectively.

Handling time showed the broadly opposite pattern. At 1 h, first-instar larvae had the longest estimated handling time (34.8 min aphid^−1^), whereas adult males and females had the shortest handling times (4.8 and 3.1 min aphid^−1^, respectively). Fourth-instar larvae also had a relatively short handling time at 1 h (11.5 min aphid^−1^). Across exposure durations, attack rate estimates generally declined from 1 to 24 h, while handling time estimates increased or stabilized. This pattern is consistent with decreasing hunger-driven attack activity and increasing effects of satiation, digestion, and prey depletion during longer exposures. For late-instar larvae and adults, the 6 h estimates provided the clearest intermediate comparison because they included sustained foraging but were less affected than the 24 h estimates by near-complete prey depletion. At 6 h, fourth-instar larvae retained the highest attack rate, whereas adults showed the shortest handling times. Thus, the 6 h models support strong short-term searching and prey-processing capacity in later stages, while the 24 h estimates should not be interpreted as absolute maximum daily intake.

The generalized Rogers’ scaling exponent q did not provide consistent evidence for positive density-dependent attack rates. Among the three treatments where the generalized Rogers’ model was AIC-selected, q was positive but its confidence interval included zero for second-instar larvae at 6 h (q = 0.53, 95% CI: −0.14 to 1.21), whereas q was negative for adult males at 1 h (q = −1.01, 95% CI: −1.04 to −0.98) and adult females at 6 h (q = −0.34, 95% CI: −0.54 to −0.13). Thus, model improvement came from flexibility in density-dependent attack rate, not from consistent support for a Type III-like increase in attack rate with prey density.

## 4. Discussion

This study quantified how larvae and adults of *S. folchinii* consumed *M. persicae*. The results point to three key patterns. First, larval feeding behavior was consistent with extra-oral digestion, whereas adults chewed aphids directly. Second, the first-instar larvae could feed simultaneously on one aphid when four larvae were confined with a single prey item. Third, aphid consumption increased strongly from early larvae to late larvae and adults, and functional response models most often fitted the data as Type II-like responses within the tested density range.

The larval feeding behavior observed in this study was consistent with extra-oral digestion, a feeding mode that is common in predaceous arthropods and allows predators to liquefy and ingest prey tissues externally [29]. First- and second-instar larvae are small relative to third-instar *M. persicae* nymphs, and biting an appendage may allow them to hold the prey while removing body contents. Adults handled prey differently. They detected aphids with the maxillary palps, bit rapidly, and consumed the aphid by mastication. These differences in feeding behaviour may help explain why later stages and adults consumed aphids more quickly than early larvae. However, because our evidence was based on stereomicroscope observations, extra-oral digestion should be regarded as an inferred feeding mode in this study. Confirmation of the underlying physiological mechanism would require additional evidence, such as enzyme assays or histological examination of partially consumed prey.

The first-instar assay demonstrated prey sharing under constrained prey-limited conditions. Similar shared feeding has been reported in other ladybird larvae, and recent work has shown that prey sharing in aphid predators can be density-dependent and associated with prey mortality under some predator/prey ratios [30,31]. However, the present assay used one larval density, one prey stage, a single aphid, and a small confined arena. Therefore, these results provide evidence that early-stage larvae can share the same aphid when prey is limited in a confined arena, rather than as evidence that prey sharing is frequent or adaptive under natural aphid-colony conditions. Whether this behavior improves larval survival, development, or growth requires further investigation.

Consumption increased clearly with predator development. First-instar larvae consistently had the lowest consumption, second instars were intermediate, and third instars, fourth instars, and adults reached near-complete consumption at the highest prey density after 24 h. This pattern agrees with the general biology of aphidophagous coccinellids, in which body size, mobility, gut capacity, and prey-processing ability increase across development [5,6]. It also agrees with reports that late larvae and adults are usually the strongest immediate consumers in coccinellid-aphid systems [32,33,34]. Early larvae should therefore not be treated as equivalent to adults in short-term consumption models, although their contribution may become important through persistence, recruitment, and group-feeding behavior. Because third- and fourth-instar larvae and adults nearly depleted the highest aphid density provided, the 24 h endpoint should be interpreted as localized patch-clearing capacity within the tested seedling micro-arena, rather than as an estimate of maximum daily consumption. Larger host plants and higher aphid densities will be required to estimate the upper asymptote of consumption more reliably. 

The functional-response results should be considered Type II-like, but not as a formal Type II diagnosis for every stage and exposure duration. Logistic regression diagnosed Type II responses only for adult females and adult males at 1 h. For larval stages and longer adult exposures, logistic regression did not clearly assign response type; these non-significant results should be interpreted as inconclusive rather than as evidence against a Type II-like response, because limited replication, the restricted prey-density range, and near-complete prey depletion in some treatments likely limited the diagnostic power of logistic regression. In contrast, AIC-based model comparison most often selected Rogers’ Type II or a closely related generalized Rogers model. The two analyses answer different questions. Logistic regression tests whether proportional consumption changes with prey density, whereas AIC comparison explains which model best fits the observed depletion data [24,26]. Therefore, *S. folchinii* showed mostly Type II-like functional responses under the tested arena conditions.

The predominance of Type II-like responses in *S. folchinii* is consistent with the broader functional-response literature on aphidophagous coccinellids. Type II responses are common in arthropod predators and are characterized by high proportional consumption at low prey densities followed by declining proportional consumption as handling, digestion, or satiation constrain intake [20,23,24]. In practice, this pattern is most relevant to early aphid colonies, where prey numbers may still fall within the predator’s handling capacity [22,23]. Similar Type II or Type II-like responses have been reported in several ladybird–aphid systems, and previous studies have shown that prey consumption, attack rate, and handling time can vary with predator stage, sex, prey species, and experimental conditions [27,28]. Comparable patterns have also been reported for other aphidophagous coccinellids, including *Scymnus syriacus* feeding on aphids [34,35]. The present results therefore place *S. folchinii* within a broader coccinellid pattern, while adding stage-specific evidence for a small scymnine coccinellid whose predatory capacity has not been experimentally characterized.

In this study, parameter estimates changed with exposure duration. Attack rates were generally highest at 1 h and lower at 24 h, while handling times increased or stabilized over longer exposures. This does not necessarily indicate that predators’ searching efficiency declined over time. Rather, short assays capture the initial hunger-driven phase, whereas longer assays incorporate prey depletion, satiation, digestion, resting, and reduced foraging motivation. Handling time in functional response models can include prey capture, subjugation, ingestion, digestion, and post-feeding inactivity [20,36]. For a small predator feeding on soft-bodied aphids, digestive constraints may be especially important during extended exposures. Although the generalized Rogers’ model was selected in three treatments, the scaling exponent q did not consistently support Type III-like density dependence. The slight AIC advantage of the generalized model in these cases probably reflects minor density-dependent variation in attack efficiency rather than a biologically consistent shift toward a sigmoidal response. Thus, the results are best interpreted as predominantly Type II-like predation with limited context-dependent variation in attack rate [25,37].

The seedling micro-arena was more realistic than a bare Petri dish, but it was still a simplified laboratory arena. Plant architecture, arena size, prey spatial distribution, predator experience, alternative prey, microclimate, and interactions with other natural enemies can all influence realized functional responses [38,39,40,41,42,43]. The small arena may have increased predator–prey encounter rates at higher aphid densities, while the maximum density of 16 aphids was insufficient to fully resolve the upper asymptote for late-instar larvae and adults, which consumed nearly all available prey after 24 h. Therefore, the 24 h endpoint is best interpreted as a measure of localized patch-clearing capacity rather than maximum physiological consumption. The 12 h starvation period was used to standardize hunger before the assays; however, because of their smaller body size and lower energetic reserves, first-instar larvae may have been more sensitive to this treatment than later instars or adults. Therefore, stage-specific comparisons involving first instars should be interpreted with this potential limitation. Future assays should include higher prey densities and/or larger plant arenas to better estimate maximum daily consumption under field conditions.

For biological control, the present results support further evaluation of *S. folchinii* as a potential predator of early aphid colonies, rather than as evidence for suppression of established high-density infestations. Late-instar larvae and adults are likely to provide the strongest immediate suppression, whereas early instars may contribute more gradually through sustained feeding and prey sharing when larvae co-occur under prey-limited conditions. This stage-dependent interpretation is important because aphid management in crop systems is shaped by several factors, including insecticide resistance, host-plant structure, prey distribution, and interactions among natural enemies. Therefore, the laboratory patterns reported here should be tested under greenhouse or semi-field conditions using larger host plants, mixed aphid stages, variable predator densities, and realistic prey aggregation [1,2,4,44,45]. Recent studies similarly show that ladybird-mediated aphid control depends on ecological context [46], species-specific predation and competition [47], and predator developmental stage [48]. Future work should therefore test *S. folchinii* under greenhouse or semi-field conditions. These studies should include larger host plants, mixed aphid stages, mated and older adults, variable predator density, and realistic prey aggregation. Longer-term studies are also needed, including compatibility with parasitoids and other predators.

Overall, *S. folchinii* showed clear stage-dependent aphid consumption, larval feeding behavior consistent with extra-oral digestion, and mostly Type II-like predation patterns under the tested conditions. These findings provide baseline laboratory evidence supporting further evaluation of this small-bodied coccinellid as a potential predator of early aphid colonies in conservation or augmentative biological control. Still, future greenhouse and field validation are needed before operational recommendations can be made.

## Figures and Tables

**Table 1:** Type III Wald χ^2^ tests for aphid consumption models of *S. folchinii* feeding on third-instar *M. persicae* nymphs.

Response/Model	Fixed Effect	Wald χ^2^	df	*p*-Value
	Predator stage	49.70	5	<0.001
	Exposure time	127.79	2	<0.001
	Initial aphid density	101.65	1	<0.001
Cumulative consumption, 1–24 h	Predator stage × exposure time	22.76	10	0.0117
	Predator stage × initial aphid density	11.68	5	0.0394
	Exposure time × initial aphid density	25.89	2	<0.001
	Predator stage × exposure time × initial aphid density	17.67	10	0.0609
	Predator stage	12.41	5	0.0296
Twenty-four-hour consumption	Initial aphid density	16.38	1	<0.001
	Predator stage × initial aphid density	1.65	5	0.895
	Adult sex	0.14	1	0.713
Adult 24 h consumption	Initial aphid density	3.14	1	0.076
	Adult sex × initial aphid density	0.09	1	0.765

Note: Cumulative consumption was analyzed using a repeated-measures beta-binomial mixed model with predator identity as a random intercept. Twenty-four-hour consumption was analyzed using beta-binomial endpoint models. Initial aphid density was included as a log_2_-transformed continuous predictor.

**Table 2:** Logistic regression diagnosis and AIC-best mechanistic model for the functional response of *S. folchinii* feeding on *M. persicae.*

Stage	Duration (h)	P_1_	*p*	Logistic Diagnosis	AIC-Best Model
1st instar	1	−0.594	0.373	Not determined	Rogers’ Type II
	6	−0.020	0.971	Not determined	Rogers’ Type II
	24	0.513	0.430	Not determined	Rogers’ Type II
	1	0.378	0.534	Not determined	Rogers’ Type II
2nd instar	6	1.059	0.155	Not determined	Generalized Rogers’ model with a scaling exponent
	24	1.496	0.244	Not determined	Rogers’ Type II
	1	−0.603	0.292	Not determined	Rogers’ Type II
3rd instar	6	0.520	0.507	Not determined	Rogers’ Type II
	24	1.495	0.472	Not determined	Rogers’ Type II
	1	−0.180	0.767	Not determined	Rogers’ Type II
4th instar	6	−25.208	0.999	Not determined	Rogers’ Type II
	24	−25.260	0.999	Not determined	Rogers’ Type II
	1	−1.242	0.037	Type II	Rogers’ Type II
Adult female	6	−1.199	0.256	Not determined	Generalized Rogers’ model with a scaling exponent
	24	−0.343	0.838	Not determined	Rogers’ Type II
	1	−1.453	0.012	Type II	Generalized Rogers’ model with a scaling exponent
Adult male	6	−0.621	0.449	Not determined	Rogers’ Type II
	24	1.227	0.562	Not determined	Rogers’ Type II

Note: P_1_ is the first-order coefficient from logistic regression of proportional prey consumption as a function of initial prey density. A significantly negative P_1_ indicates Type II; a significantly positive P_1_ followed by a negative quadratic term indicates Type III; otherwise, the response type is reported as not resolved by logistic regression. ‘Not determined’ indicates that the response type was not resolved by logistic regression and should not be interpreted as evidence against a Type II-like response. Large coefficient estimates with non-significant *p*-values indicate unstable logistic fits under near-complete consumption and should not be interpreted biologically.

**Table 3:** Rogers’ Type II parameter estimates and generalized Rogers’ scaling exponent for *S. folchinii* feeding on *M*. *persicae*.

Stage	Duration (h)	*a* (d^−1^; 95% CI)	*T_h_* (min aphid^−1^; 95% CI)	*q* (95% CI)
	1	9.00 (3.34 to 24.26)	34.8 (15.7 to 77.2)	−0.52 (−0.88 to −0.17)
1st instar	6	4.50 (2.45 to 8.28)	107.0 (71.3 to 160.7)	−0.05 (−1.31 to 1.21)
	24	1.97 (1.32 to 2.93)	175.5 (127.1 to 242.5)	0.13 (−0.64 to 0.90)
	1	8.80 (5.02 to 15.42)	7.8 (2.1 to 29.2)	−0.04 (−1.09 to 1.02)
2nd instar	6	10.00 (7.06 to 14.18)	35.0 (26.4 to 46.2)	0.53 (−0.14 to 1.21)
	24	3.83 (2.75 to 5.36)	85.3 (64.6 to 112.6)	0.29 (−0.18 to 0.76)
	1	15.38 (8.40 to 28.15)	14.2 (7.6 to 26.5)	−0.42 (−1.54 to 0.69)
3rd instar	6	10.55 (7.58 to 14.68)	28.4 (21.2 to 38.1)	0.33 (−0.24 to 0.90)
	24	4.03 (2.77 to 5.89)	35.5 (15.2 to 83.1)	−0.08 (−0.80 to 0.63)
	1	45.63 (28.39 to 73.34)	11.5 (8.5 to 15.7)	0.42 (−0.60 to 1.44)
4th instar	6	34.27 (20.55 to 57.13)	35.2 (30.0 to 41.4)	0.46 (−0.30 to 1.22)
	24	4.25 (2.85 to 6.35)	31.7 (11.4 to 88.5)	−0.25 (−0.55 to 0.05)
	1	24.83 (16.96 to 36.34)	4.8 (2.6 to 9.0)	−0.24 (−1.04 to 0.55)
Adult female	6	8.26 (5.98 to 11.41)	15.0 (8.3 to 27.4)	−0.34 (−0.54 to −0.13)
	24	4.18 (2.86 to 6.11)	38.3 (18.0 to 81.4)	0.17 (−0.44 to 0.78)
	1	19.90 (13.82 to 28.67)	3.1 (1.1 to 8.7)	−1.01 (−1.04 to −0.98)
Adult male	6	7.59 (5.54 to 10.39)	11.4 (5.1 to 25.9)	−0.24 (−0.43 to −0.04)
	24	4.69 (3.12 to 7.05)	43.2 (22.7 to 82.1)	0.17 (−0.43 to 0.78)

Note: *a* and *T_h_* are attack rate and handling time estimated from the Rogers’ Type II model. Because exposure duration was modeled in days and prey availability was expressed as number of aphids per seedling micro-arena, a is reported as d^−1^ for the fixed seedling micro-arena used in this experiment. $T_{h}$ was converted from days to minutes per aphid for readability. q is the dimensionless scaling exponent from the generalized Rogers’ model; q = 0 corresponds to the standard Rogers’ Type II model. Confidence intervals are bootstrap 95% CIs. For late-instar larvae and adults, 24 h estimates should be interpreted cautiously because prey availability at the highest density was nearly exhausted; these estimates describe model-fitted depletion dynamics within the tested density range rather than absolute physiological maxima.

## Data Availability

The original contributions presented in the study are included in the article. Further inquiries can be directed to the corresponding author.

## Appendix A

**Table A1:** Type III Wald χ^2^ tests for the conditional interval beta-binomial model of aphid consumption by *S. folchinii.*

Fixed Effect	Wald χ^2^	df	*p*-Value
Predator stage	12.90	5	0.0244
Observation interval	41.77	2	<0.001
Initial aphid density	14.03	1	<0.001
Predator stage × observation interval	34.43	10	<0.001
Predator stage × initial aphid density	2.73	5	0.742
Observation interval × initial aphid density	28.77	2	<0.001
Predator stage × observation interval × initial aphid density	28.17	10	0.0017

Note. Interval-specific aphid consumption was analyzed using a conditional beta-binomial mixed model. Observation intervals were 0–1 h, 1–6 h, and 6–24 h. Initial aphid density was included as a log_2_-transformed continuous predictor. Predator identity was included as a random intercept.

**Table A2:** Comparison of candidate functional response models fitted to *S. folchinii* preying on *M. persicae* across developmental stages and exposure durations.

Stage	Duration (h)	Model	k	AIC	ΔAIC	*w_i_*	Rank
1st instar	1	Rogers’ Type II	2	231.96	0.00	0.529	1
	1	Generalized Rogers’ (flexpnr)	3	233.00	1.04	0.314	2
	1	Hassell Type III	4	235.21	3.25	0.104	3
	1	Holling Type I	1	236.57	4.61	0.053	4
	6	Rogers’ Type II	2	343.97	0.00	0.665	1
	6	Generalized Rogers’ (flexpnr)	3	345.96	1.99	0.245	2
	6	Hassell Type III	4	347.97	4.00	0.090	3
	6	Holling Type I	1	366.18	22.21	0.000	4
	24	Rogers’ Type II	2	403.70	0.00	0.656	1
	24	Generalized Rogers’ (flexpnr)	3	405.59	1.89	0.255	2
	24	Hassell Type III	4	407.70	4.00	0.089	3
	24	Holling Type I	1	431.29	27.59	0.000	4
	1	Rogers’ Type II	2	333.14	0.00	0.402	1
	1	Holling Type I	1	333.18	0.03	0.395	2
	1	Generalized Rogers’ (flexpnr)	3	335.14	2.00	0.148	3
	1	Hassell Type III	4	337.13	3.98	0.055	4
	6	Generalized Rogers’ (flexpnr)	3	382.06	0.00	0.471	1
	6	Rogers’ Type II	2	382.66	0.60	0.348	2
2nd instar	6	Hassell Type III	4	383.97	1.91	0.181	3
	6	Holling Type I	1	415.81	33.75	0.000	4
	24	Rogers’ Type II	2	275.71	0.00	0.548	1
	24	Generalized Rogers’ (flexpnr)	3	276.37	0.65	0.395	2
	24	Hassell Type III	4	280.26	4.55	0.056	3
	24	Holling Type I	1	300.83	25.12	0.000	4
	1	Rogers’ Type II	2	341.81	0.00	0.598	1
	1	Generalized Rogers’ (flexpnr)	3	343.04	1.23	0.324	2
	1	Hassell Type III	4	346.23	4.42	0.066	3
	1	Holling Type I	1	349.54	7.73	0.013	4
	6	Rogers’ Type II	2	365.50	0.00	0.507	1
	6	Generalized Rogers’ (flexpnr)	3	366.21	0.71	0.355	2
	6	Hassell Type III	4	368.09	2.59	0.139	3
3rd instar	6	Holling Type I	1	393.32	27.83	0.000	4
	24	Rogers’ Type II	2	132.31	0.00	0.667	1
	24	Generalized Rogers’ (flexpnr)	3	134.25	1.94	0.252	2
	24	Hassell Type III	4	136.52	4.21	0.081	3
	24	Holling Type I	1	550.02	417.70	0.000	4
	1	Rogers’ Type II	2	386.00	0.00	0.596	1
	1	Generalized Rogers’ (flexpnr)	3	387.27	1.28	0.315	2
	1	Hassell Type III	4	389.81	3.81	0.089	3
	1	Holling Type I	1	421.58	35.58	0.000	4
	6	Rogers’ Type II	2	269.51	0.00	0.526	1
	6	Generalized Rogers’ (flexpnr)	3	270.49	0.99	0.321	2
4th instar	6	Hassell Type III	4	271.98	2.47	0.153	3
	6	Holling Type I	1	351.74	82.24	0.000	4
	24	Rogers’ Type II	2	109.45	0.00	0.602	1
	24	Generalized Rogers’ (flexpnr)	3	110.77	1.32	0.311	2
	24	Hassell Type III	4	113.31	3.86	0.087	3
	24	Holling Type I	1	421.07	311.62	0.000	4
	1	Rogers’ Type II	2	424.36	0.00	0.651	1
	1	Generalized Rogers’ (flexpnr)	3	425.95	1.59	0.294	2
	1	Holling Type I	1	430.46	6.10	0.031	3
	1	Hassell Type III	4	430.95	6.59	0.024	4
	6	Generalized Rogers’ (flexpnr)	3	349.27	0.00	0.618	1
	6	Rogers’ Type II	2	350.35	1.08	0.360	2
Adult female	6	Holling Type I	1	356.03	6.76	0.021	3
	6	Hassell Type III	4	362.21	12.94	0.001	4
	24	Rogers’ Type II	2	131.57	0.00	0.628	1
	24	Generalized Rogers’ (flexpnr)	3	133.30	1.73	0.265	2
	24	Hassell Type III	4	135.11	3.54	0.107	3
	24	Holling Type I	1	550.02	418.45	0.000	4
	1	Generalized Rogers’ (flexpnr)	3	424.96	0.00	0.785	1
	1	Rogers’ Type II	2	428.79	3.83	0.116	2
	1	Holling Type I	1	429.89	4.93	0.067	3
	1	Hassell Type III	4	431.30	6.34	0.033	4
	6	Rogers’ Type II	2	347.17	0.00	0.490	1
	6	Generalized Rogers’ (flexpnr)	3	347.82	0.65	0.354	2
Adult male	6	Holling Type I	1	349.54	2.38	0.149	3
	6	Hassell Type III	4	355.73	8.57	0.007	4
	24	Rogers’ Type II	2	118.76	0.00	0.632	1
	24	Generalized Rogers’ (flexpnr)	3	120.48	1.72	0.268	2
	24	Hassell Type III	4	122.44	3.68	0.100	3
	24	Holling Type I	1	485.54	366.78	0.000	4

Notes: k, number of estimable parameters; AIC, Akaike’s information criterion; ΔAIC, difference between the AIC of each model and the lowest AIC within each stage × duration combination; *w_i_*, Akaike weight. Models with ΔAIC < 2 were considered to have substantial support. The Generalized Rogers’ model (flexpnr) allows density-dependent attack rate and should not be interpreted as a discrete Type III model.
